# Efficacy of RUTI^®^ immunotherapy against active tuberculosis in a mouse model challenges the Koch phenomenon

**DOI:** 10.3389/ftubr.2023.1240684

**Published:** 2023-11-08

**Authors:** Pablo Soldevilla, Anna Buisan, Jorge Diaz, Sergi Saladrigas, Yaiza Rosales, Lilibeth Arias, Alexandra Jimenez-Melsio, Cristina Vilaplana, Merce Amat Fabregat, Pere-Joan Cardona

**Affiliations:** ^1^Unitat de Tuberculosi Experimental, Germans Trias i Pujol Research Institute (IGTP), Badalona, Spain; ^2^Servei de Microbiologia, Laboratori Clínic de la Metropolitana Nord (LCMN), Hospital Universitari Germans Trias i Pujol, Badalona, Spain; ^3^Centro de Investigación Biomédica en Red de Enfermedades Respiratorias (CIBERES), Madrid, Spain; ^4^Department of Genetics and Microbiology, Autonomous University of Barcelona, Cerdanyola del Vallès, Spain; ^5^Archivel Farma, s.l., Badalona, Spain; ^6^Centre de Medicina Comparativa i Bioimatge de Catalunya (CMCiB), Badalona, Spain

**Keywords:** *Mycobacterium tuberculosis*, chemotherapy, RUTI^®^, immunotherapy, Koch phenomenon

## Abstract

**Objectives:**

The objective of this study was to evaluate the safety and efficacy of the therapeutic vaccine RUTI^®^ with or without the standard of care (SOC) chemotherapy for TB in an experimental murine model.

**Methods:**

We assessed the efficacy of RUTI^®^, a vaccine based on pasteurized and freeze-dried cell-wall fragments from *Mycobacterium tuberculosis* (Mtb), the SOC for sensitive TB (isoniazid, rifampicin, ethambutol, and pyrazinamide) in the murine active TB model in C3HeB/FeJ strain (Kramnik model). We evaluated the bacillary load in the lungs and spleen, the immune response against specific Mtb antigens (PPD, HSP16.3, ESAT-6, and PsTS1), and lung damage in paraffined tissues for qualitative and quantitative analysis.

**Results:**

RUTI^®^ significantly reduces the pulmonary damage (x3) and the bacterial burden in the lungs (1.5 log10) and spleen (1 log10), and maintains the cellular immune response against ESAT-6, compared to SOC. There was also an additive effect when administered in combination with SOC, increasing the reduction of the lung damage (x2), the bacillary load in lungs (1 log10) and increasing the immune response against PPD, HSP16.3 and PsTS1.

**Discussion:**

Therapeutic vaccination against TB has been avoided for decades due to fear of toxicity through the Koch phenomenon. These data show for the first time the safety of immunotherapy with Mtb antigens in an active TB model, adding efficacy to SOC. This encourages the beginning of clinical studies to evaluate the safety and efficacy of RUTI^®^ in TB patients to improve their health, reduce its potential infectiousness, and reduce the length of treatment.

## Background

Currently, tuberculosis (TB) is still one of the deadliest infectious diseases, and more so as a consequence of the COVID-19 pandemic, which has seriously hampered its control. Despite recent efforts to increase the chemotherapy pipeline, there are still two major problems to address, specifically the length of therapy and the increase in multidrug resistance.

Tuberculin, created by Robert Koch, was the first therapeutic vaccine composed of *Mycobacterium tuberculosis* (Mtb) antigens to target incipient TB cases. However, since the first clinical trial run in 1891 on a wide TB clinical spectrum, it has been claimed to produce extensive inflammation and reactivation of tuberculosis lesions. This toxic reaction was named the Koch Phenomenon (1, 2) and since then, the therapeutic use of Mtb antigens has been avoided. But recently, therapeutic vaccines have regained some consideration, as they have the potential to be administered as a complement to the antibiotic treatment against TB. In fact, the WHO elaborated a list of considerations regarding therapeutic vaccination against TB, where they consider that one of the targets of these vaccines should be to shorten the chemotherapy (3). Animal models are an efficient tool to study the effect of new anti-TB drugs and treatments at every stage of the disease, each one with different characteristics on Mtb populations and immune response (4). Two common laboratory animals used for that purpose are guinea pigs and mice. Guinea pigs develop large necrotic lesions, but rarely develop liquefaction (5), which is a key aspect of active TB lesions in humans. On the other hand, mice show greater tolerance to Mtb infection than guinea pigs. That is why they are frequently used to study the latent stage of the disease (6). Nevertheless, the C3HeB/FeJ mouse strain is able to develop liquefacted human-like lesions and is thus considered a feasible active TB model (7). Previous studies in mice have shown positive results after combining chemotherapy and therapeutic vaccination (8–10), but to our knowledge, there are no assays performed in the model of active TB in C3HeB/FeJ mice.

RUTI^®^ is a liposomal suspension of the drug substance (DS) with sucrose as a charge excipient. Designed to be administered as a therapeutic vaccine to shorten TB chemotherapy. The DS consists of purified, pasteurized, and freeze-dried 100 nm cell-wall fragments from Mtb (MtbFCW) grown under stress conditions (i.e., starvation, low pO_2_, and low pH) to mimic the hostile environment of a granuloma that leads Mtb to a non-replicating status or dormancy. Thus, the DS contains multiple antigens, classically described as representative of replicating and non-replicating Mtb, that are able to boost a Th1 response and reduce the bacillary load in models of latent tuberculosis infection (LTBI) in laboratory animals (11, 12). RUTI^®^ is able to increase the frequency of non-classical Ly6C- monocytes, promoting an effective cell-mediated response while limiting excessive inflammation (13). Additionally, RUTI^®^ has been already tested in clinical trials. In particular, a phase I study in healthy subjects (14) and a phase II study in patients with LTBI (15) showed good safety, tolerability, and immunogenicity. After studying the vaccine in LTBI, we wanted to assess the activity of RUTI^®^ against active TB. We tested the safety and efficacy of RUTI^®^ in an experimental murine model of active TB in the mouse strain C3HeB/FeJ, when administered alone, or in combination with the standard of care (SOC) chemotherapy.

## Materials and methods

C3HeB/FeJ mice (6–8 weeks old) were obtained from the Jackson Laboratory and housed in a Biosafety Level 3 (BSL-3) facility. The procedure was reviewed by the Animal Ethics Committee of the Germans Trias i Pujol Research Institute (IGTP) and by the Department of Environment of the Catalan Government (IGTP.000037-20 8CA0). The mice were monitored daily following a strict monitoring protocol in order to ensure animal welfare and euthanized if required (i.e., weight loss over 25% based on a clinical score that includes response to handling, physical attitude, breathing signs, aspect of feces, and neurological signs). Euthanasia for the entire project was performed using isoflurane (inhalation overdose) followed by cervical dislocation.

Animals were randomly distributed between four groups: animals treated with sham (control group), only treated with isoniazid, rifampicin, ethambutol, and pyrazinamide (HRZE) (chemotherapy group), only treated with RUTI^®^ (RUTI group) and treated with HRZE plus RUTI^®^ (combined group). Each group contained the same number of males and females (*n* = 6) and the experiment was repeated twice. All animals were intravenously infected via lateral tail vein with 2 × 10^4^ Colony Forming Units (CFUs) of Mtb H37Rv strain Sanger in a final volume of 0.2 mL per animal. The infection was left to evolve for a period of 5 weeks, the time when liquefacted human-like lesions develop. At this time, we started the treatment.

Animals treated with SOC chemotherapy received a commercial combination of HZRE (RIMSTAR^®^, Sandoz Farmaceutica, Barcelona) adjusted to their weight daily at the beginning of the fifth week after the infection orally through gavage. RUTI^®^ (Archivel Farma s.l., Badalona, Spain) vaccine was administered subcutaneously at a dose of 200 ug MtbFCW only once at the beginning of the fifth week.

The control group was euthanized on day 35 (week 5). The rest of the groups were euthanized on day 42, after finishing the treatments (week 6). The left lung and half of the spleen were removed from each animal, homogenized, plated in Middlebrook 7H11 agar plates (BD Diagnostics, Spark, USA), and incubated at 37°C for 28 days, when the bacillary load was measured in CFUs. The lower right lung lobe was embedded in paraffin, sliced, and stained with hematoxylin-eosin (4 recuts per animal). The pulmonary damaged area was calculated with image analysis software (NISELements D version 3.0x) using standard procedures (16). The other half of the spleen was used for splenocyte isolation to perform an ELISpot assay to measure the activated antigen-specific IFN-γ secreting T cells response against PPD, at a final concentration of 30μg/mL, and against ESAT-6, HSP16.3, and PsTS1 (Lionex, Braunschweig, Germany) at a final concentration of 10μg/ml using standard procedures. The spot-forming units (SFU) were counted using an ELISPOT reader and presented as the number of spots minus the number of spots obtained with each corresponding blank. Negative values have been expressed as “0” (17).

Statistical analysis: Animal number per group was determined according to previous experience (16). The nonparametric Mann-Whitney test was used to compare groups in bacillary load, pathology, and immune response, using GraphPad Prism (GraphPad software v7.0, La Jolla, California, USA).

## Results

**Figure 1:**
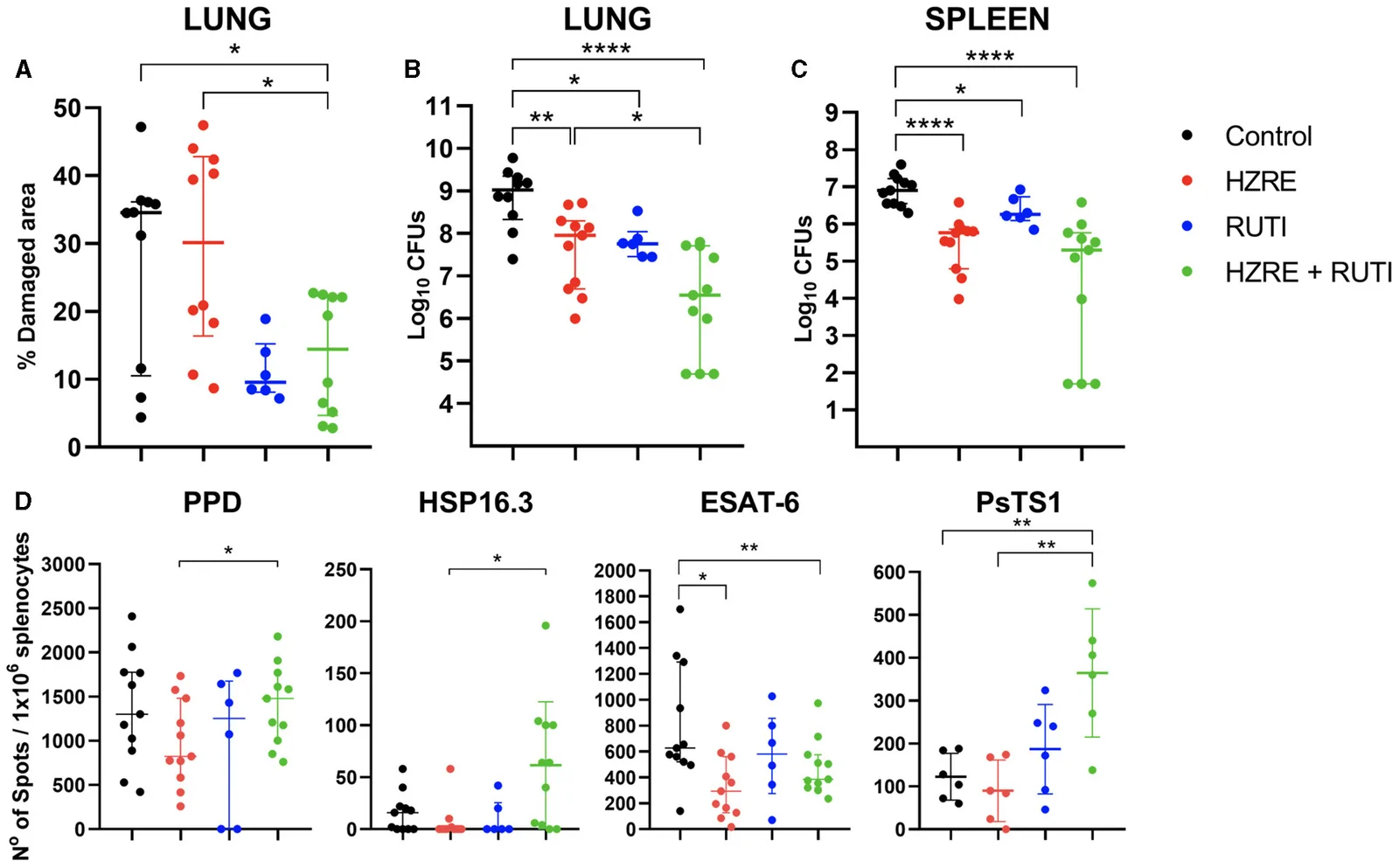
Evaluation of pathology, bacillary load, and immune response. **(A)** Pulmonary damage registered for each group. Bacterial burden is shown in **(B)** for the lungs and **(C)** for the spleen. **(D)** ELISPOT results after stimulating splenocytes with PPD, HSP16.3, ESAT-6, and PsTS1. Each dot represents one animal. The limit of detection is one spot. Mann Whitney test (*p < 0.05, **p < 0.01, ****p < 0.001). These data represent the outcomes of pooled data from two independent experiments.

RUTI^®^ vaccine was safe and well tolerated in vaccinated animals, with no toxic effects registered during the assay (Supplementary Figure 1). The vaccine alone was able to reduce the damaged pulmonary area, although not significantly when compared with the control and HZRE groups. However, RUTI^®^ had a positive additive effect when given with HRZE, as the combination significantly reduced the damaged area, while chemotherapy alone had no effect on this parameter (Figure 1A). In the animals treated with RUTI^®^, with or without HRZE, the bacillary load in the lungs and spleen was reduced significantly compared to the control group (Figures 1B, C, respectively).

Looking at the immune response, and in contrast with the HRZE treatment, vaccination alone maintained the immunity against ESAT-6, even when reducing the bacillary load at a similar level. However, the combination of both therapies significantly increased the immune response against all antigens except for ESAT-6, when compared with chemotherapy alone (Figure 1D).

**Figure 2:**
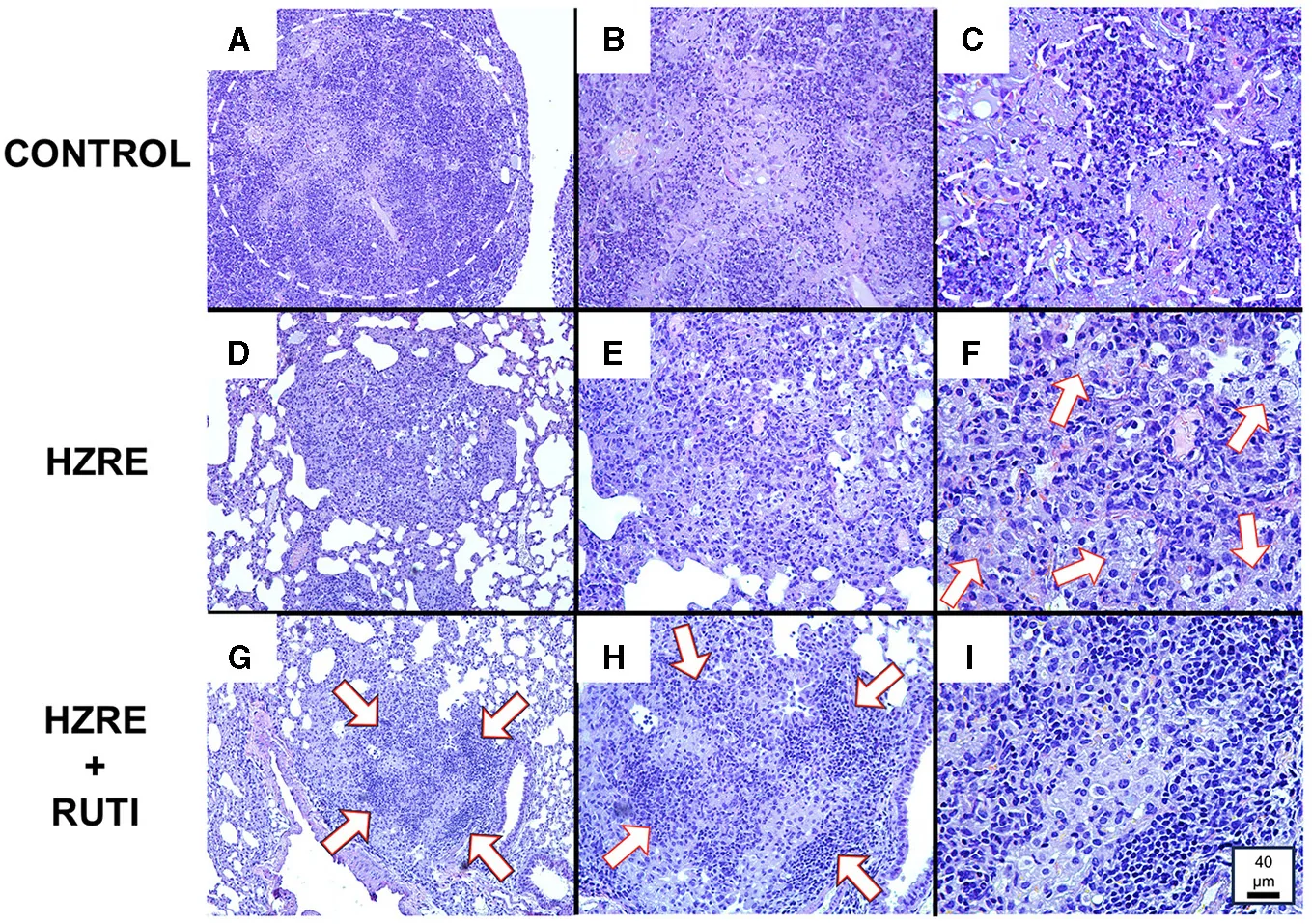
Histological analysis. **(A–C)** Show a lesion from a mouse of the control group at 10X, 20X, and 40X magnification, respectively. Similarly, **(D–F)** show a lesion from the chemotherapy group; and **(G–I)** show a lesion from the chemotherapy plus RUTI group. Animals from the control group presented bigger necrotic lesions [**(A)** encircled area] in a pre-liquefaction phase [**(C)** outside of the marked area], and with the presence of apoptotic and necrotic neutrophils [**(C)** inside part of the marked area]. Animals treated with chemotherapy presented lesions filled mainly with macrophages, both alveolar and foamy (white arrows) **(F)**, while the chemotherapy plus RUTI group showed structured granulomas with increased presence of lymphocytic clumps (white arrows).

Histometric analysis also revealed qualitative differences in the cellular composition of the lesions. The animals in the control group developed necrotic lesions, filled with apoptotic and necrotic neutrophils. At the moment of the euthanasia, most of the lesions showed initial stages of liquefaction (Figures 2A–C). While the chemotherapy group presented an infiltration based on foamy and alveolar macrophages (Figures 2D–F), the group treated with chemotherapy and RUTI^®^ showed an increased lymphocytic infiltration in the granulomas (Figures 2G–I).

## Discussion

This assay is the first one that demonstrated that immunotherapy of experimental active TB with Mtb antigens does not generate a damaging exacerbated immune response. On the contrary, it is able to reduce the pathology and the bacillary load, showing an additive effect when it is administered together with first-line SOC anti-TB drugs.

The short-term TB chemotherapy initiated in the 1980s was a great achievement as it provided a strong and successful treatment (18). But the negative aspects should be underlined. These are the length of the treatment, which is a barrier to compliance, and the slow reduction of the bacillary load in patients, who maintain the capacity to further disseminate the infection during the first weeks of chemotherapy. The latter is of paramount importance in countries with few resources, where patient containment is challenging (19). That's why our experiments were focused in this period, together with the fact that it is precisely in this phase, where there is the highest bacillary load when it was expected the highest toxicity of immunotherapy (20).

Our data has shown how chemotherapy can modulate and interfere with the innate immune response; an aspect that has been very little studied in the case of TB (21). In this regard, we have seen how animals treated only with chemotherapy kept a higher presence of foamy macrophages in the lesions in comparison with animals treated with chemotherapy plus RUTI^®^. This observation acquires importance when we consider that foamy macrophages have been associated with the dissemination of Mtb through the respiratory system (22). Furthermore, studies with *Salmonella* and *Chlamydia* infections have shown that chemotherapy reduces the generation of Th1 immune memory (23).

We have observed two different mechanisms in RUTI^®^ vaccination that can explain its activity. On one hand, it keeps the immune response against ESAT-6, as has been previously demonstrated (24), a response linked to the presence of active growing bacilli (25), which attends to the reduction of the bacillary load and means that it keeps the surveillance against this type of bacilli, stopping the emergence of new lesions, a fact supported by the reduction of the granulomatous infiltration. On the other hand, when given in combination with HRZE, RUTI^®^ increases the immunity against antigens related to dormant bacilli (HSP16.3 and PsTS1) (26), thus enhancing the surveillance against this bacillary population. This effect is reinforced by the histologic analysis, which shows an increase in the lymphocytic infiltration of the lesions, probably as a result of the proliferation of lymphocytes specific for HSP16.3 and PsTS1. This finding merits further investigations in order to refine the analysis of these mechanisms.

Mice treated with chemotherapy plus RUTI^®^ reduced both damaged pulmonary area and pulmonary bacterial burden and increased cellular response against *M. tuberculosis* antigens. These results show RUTI^®^ as an adjunct therapy for active TB chemotherapy administration, further improving a drastic reduction of the bacillary load at the beginning of the treatment. This is an unprecedented proof of concept that could make an important contribution to an early elimination of the bacillary load and thus to a reduction in the duration of the treatment, one of the targets defined by the WHO regarding TB therapeutic vaccines. This data encourages further assays to test new therapeutic strategies and their evaluation in human TB patients.

## Data availability statement

The raw data supporting the conclusions of this article will be made available by the authors, without undue reservation.

## Ethics statement

The animal study was approved by Animal Ethics Committee of the Germans Trias i Pujol Research Institute (IGTP) and by the Department of Environment of the Catalan Government (IGTP.000037-20 8CA0). The study was conducted in accordance with the local legislation and institutional requirements.

## Author contributions

Conceptualization, validation, and writing—review & editing: MA, CV, and P-JC. Methodology: PS, AB, JD, SS, YR, LA, AJ-M, MA, and P-JC. Software: PS and LA. Formal analysis: P-JC. Investigation, resources, supervision, and project administration: MA and P-JC. Data curation: PS, SS, MA, and P-JC. Writing—original draft preparation: PS, MA, and P-JC. Visualization: PS and P-JC. All authors have read and agreed to the published version of the manuscript.
